# Integrating Single-cell RNA-seq to construct a Neutrophil prognostic model for predicting immune responses in non-small cell lung cancer

**DOI:** 10.1186/s12967-022-03723-x

**Published:** 2022-11-18

**Authors:** Jianyu Pang, Qian Yu, Yongzhi Chen, Hongjun Yuan, Miaomiao Sheng, Wenru Tang

**Affiliations:** Laboratory of Molecular Genetics of Aging & Tumor, Medicine School, Kunming University of Science and Technology, No. 727, Jingming South Road, Kunming City, Yunnan Province China

**Keywords:** NSCLC, scRNA-seq, Tumor immune microenvironment, Neutrophil, Prognosis, Immunotherapy response

## Abstract

Non-small cell lung cancer (NSCLC) is the most widely distributed tumor in the world, and its immunotherapy is not practical. Neutrophil is one of a tumor’s most abundant immune cell groups. This research aimed to investigate the complex communication network in the immune microenvironment (TIME) of NSCLC tumors to clarify the interaction between immune cells and tumors and establish a prognostic risk model that can predict immune response and prognosis of patients by analyzing the characteristics of Neutrophil differentiation. Integrated Single-cell RNA sequencing (scRNA-seq) data from NSCLC samples and Bulk RNA-seq were used for analysis. Twenty-eight main cell clusters were identified, and their interactions were clarified. Next, four subsets of Neutrophils with different differentiation states were found, closely related to immune regulation and metabolic pathways. Based on the ratio of four housekeeping genes (*ACTB, GAPDH, TFRC, TUBB*), six Neutrophil differentiation-related genes (NDRGs) prognostic risk models, including *MS4A7, CXCR2, CSRNP1, RETN, CD177*, and *LUCAT1*, were constructed by Elastic Net and Multivariate Cox regression, and patients’ total survival time and immunotherapy response were successfully predicted and validated in three large cohorts. Finally, the causes of the unfavorable prognosis of NSCLC caused by six prognostic genes were explored, and the small molecular compounds targeted at the anti-tumor effect of prognostic genes were screened. This study clarifies the TIME regulation network in NSCLC and emphasizes the critical role of NDRGs in predicting the prognosis of patients with NSCLC and their potential response to immunotherapy, thus providing a promising therapeutic target for NSCLC.

**Supplementary Information:**

The online version contains supplementary material available at 10.1186/s12967-022-03723-x.

## Introduction

Lung cancer is a common malignant tumor in clinics worldwide, and about 85% are non-small cell lung cancer (NSCLC) [1]. Despite the progress of various treatment methods, the 5-year survival rate of NSCLC patients is still meager [2]. Immunotherapy checkpoint inhibitors have been used for the first-line treatment of patients with advanced NSCLC [3], but the proportion of effective responders to immunotherapy only reaches 63% [4]. It can be seen that an in-depth understanding of NSCLC tumor immune microenvironment (TIME) and detection of immunosuppressive resistance are vital issues in current immunotherapy. Due to the heterogeneity of immune cells, TIME is a complex system, and the heterogeneity of immune cell infiltration is a key factor affecting the response and prognosis of NSCLC and other tumor types. Therefore, the prognosis model based on specific immune cell biomarkers can predict immune response and patient prognosis more accurately. Neutrophils play a crucial role in resisting infection and maintaining dynamic tissue balance, accounting for about 70% of the white blood cells in the human peripheral blood [5, 6]. It is worth noting that Neutrophils are also involved in the occurrence and development of cancer, which affects the initiation, growth, and metastasis of primary tumors [7–10]. Neutrophils play a role in tumor promotion and anti-tumor, including promoting tumor cell clearance and toxicity to tumor cells [11–14]. It can be seen that Neutrophils play an essential role in the immune system and cancer. Hence, Neutrophil biomarkers help detect the prognosis of NSCLC patients and the immunotherapy effect. Single-cell RNA sequencing (scRNA-seq) technology is the next generation of high-throughput sequencing technology, aiming to detect a single cell’s genetic information [15], and reveal heterogeneity between different cells. As a powerful tool for exploring TIME, scRNA-seq plays an essential role in revealing the TIME map, analyzing the fate of cells, and exploring cell interactions.

In this study, to overcome the shortcomings of the small sample, we integrated two large data sets of scRNA-seq. On this basis, we analyzed cell communication to understand the interactions between different cells. Then, the pseudotime analysis of Neutrophils was carried out to explore the different differentiation states of Neutrophils, and the genes related to Neutrophil differentiation were screened. Next, based on the proportion of four housekeeping genes, we use the Elastic Net regression algorithm and Multivariate Cox regression to construct a prognostic risk model and prove that this model is an excellent biomarker for predicting the prognosis and immunotherapy effect of NSCLC patients. Finally, based on the genes in the prediction model, we conducted functional exploration and molecular docking research to understand the performance of these genes and ways to improve them (Additional file 1: Fig. S1).

## Methods

### Data sources used for analysis

The NSCLC scRNA-seq datasets were downloaded from the GEO Database [16], including GSE131907 and GSE148071. GSE131907 dataset contains 58 lung adenocarcinomas, and GSE148071 dataset contains 42 NSCLC patient data. Bulk RNA-seq data were downloaded from the TCGA Database [17] and GEO Database, including TCGA-LUAD, TCGA-LUSC, and GSE81089. The TCGA cohort was used to analyze the cell type percentages and the test set for the prognostic model establishment. The GEO cohort was used as the validation set of the prognostic model.

### Comprehensive analysis of single cell datasets and cell cluster annotation

scRNA-seq dataset analysis was performed using the Seurat (v4.1.1) in R. First, the two large scRNA-seq datasets were integrated and batch corrected by the “IntegrateData” function. Disqualified cells were then excluded from the integrated dataset according to the following quality control criteria. (1) 500 < nFeature_RNA) < 5000; (2) 200 < nCount_RNA) < 35,000; (3) percentage.mt ≥ 10%. The result is a comprehensive dataset of 202,424 cells. Next, the analysis was performed through the standard Seurat workflow. SingleR (v1.8.1), CellMarker database [18] and PanglaoDB database [19] were used for cell type annotation. In addition, the CopyKAT (v1.0.8) was used to distinguish cancer cells from normal cells, distinguishing between aneuploid and euploid cell populations.

### Annotating cell types in bulk RNA-seq datasets

CIBERSORT is a suite of algorithms for calculating cell abundance. The algorithm calculates a non-negative gene expression matrix based on the marker gene expression of a specific cell type and finally obtains the relative proportions of various cell subsets. Here, we used as input all Marker genes of a subpopulation of cells to compare differences between different cell types in tissues and between normal and tumor tissues [20].

### Cell communication analysis

The interaction patterns between cancer cells and other cells in the tumor microenvironment were calculated using the iTAKL package (v0.1.0) in R. The top 50% of highly expressed genes were selected as an input, and their location in the cellular communication network was determined through the ligand-receptor database.

### Determining different differentiation states of cell subsets

Pseudotime trajectories of Neutrophils were constructed using the Monocle (v2.22.0). The algorithm uses machine learning techniques to arrange cells into trajectories with branch points based on a specific set of genes as input. The results explain that different clades are cell populations with different differentiation states. Here we used Gene Set Enrichment Analysis (GSEA) to perform functional enrichment analysis of cells in different states. Differential analysis was performed between branches, and the differentially expressed genes were defined as branch-dependent or state-specific genes. These Neutrophil marker genes located in different branch states were defined as Neutrophil differentiation-related genes (NDRGs). In addition, somatic mutation analysis of NDRGs was performed using the maftools (v2.10.05) in R.

### Calculate the prognostic risk model

In order to make the model prediction effect more accurate, we used the Elastic Net Regression and the Multivariate Cox Regression method to construct the prognostic risk model. The cost function of the Elastic Net Regression combines the regularization methods of Lasso Regression and Ridge Regression. The size of the penalty term was controlled by two parameters, λ, and ρ. Here we use the caret (v6.0–92) and glmnet (v4.1–4) in R to select the best ρ and λ, identify reliable prognosis-related genes, and then determine the prognosis risk model based on Multivariate Cox Regression. Finally, we tested the performance of the prognostic model using the receiver operating characteristic curve (ROC) and nomogram in R to judge the predictive accuracy of the prognostic risk model and validated the prognostic risk model’s effectiveness using the survival analysis in R.

### Immune infiltration analysis

In order to evaluate the relationship between the prognostic risk model and immune infiltration, we used the single sample Gene Set Enrichment Analysis (ssGSEA) algorithm in R to calculate the degree of immune infiltration of 28 kinds of immune cells in the TCGA cohort to observe the relationship between prognostic risk and immune infiltration [21].

### Functional research of prognostic gene

To further explore why prognostic gene lead to adverse prognosis, we defined the top 30% and bottom 30% of patients with prognostic gene expression in the TCGA cohort as overexpression and low expression groups. Then, differences between groups were analyzed, and changes in pathway activity were analyzed by gene set variation analysis (GSVA).

### Drug screened and docking

Based on functional studies of six prognostic genes, we screened five protein-coding genes in addition to *LUCAT1* for targeted drugs. Drug selection criteria focused on the expression of prognostic genes in cancer patients, namely increased mRNA expression of *MS4A7*, *CXCR2*, *RETN*, and *CSRNP1*. Since *CD177* is known to be associated with neutrophils and immune prognosis, targeted drugs that promote *CD177* expression were selected. We used Autodock (Linux, v4.2) for molecular docking to study small molecules compound interacting with prognostic genes. Firstly, we downloaded the catalog of small molecules that interacted with prognostic genes from the CTD Database [22], followed by the small molecule structures from the PubChem Database [23]. Next, we searched and downloaded the biological macromolecular structures translated by the prognostic genes from the Uniport Database [24]. Finally, the automatic docking of biological macromolecules and small molecular compounds is carried out according to the standard docking process, and the small molecule with the substantial interaction with the biological macromolecules is determined by the lowest binding energy. Moreover, visualize the results by PyMol (v2.6, Open-Source).

### Statistical analysis

R version 4.1.1 was used for statistical analysis. Nonparametric tests were used for statistical tests between different groups, and log rank test was used to test for significant differences in survival probability between samples, with *P-value* < 0.05 indicated statistical significance. Spearman Rank Correlation Analysis was used to calculate correlations.

## Results

### Identification of cell types

**Fig. 1:**
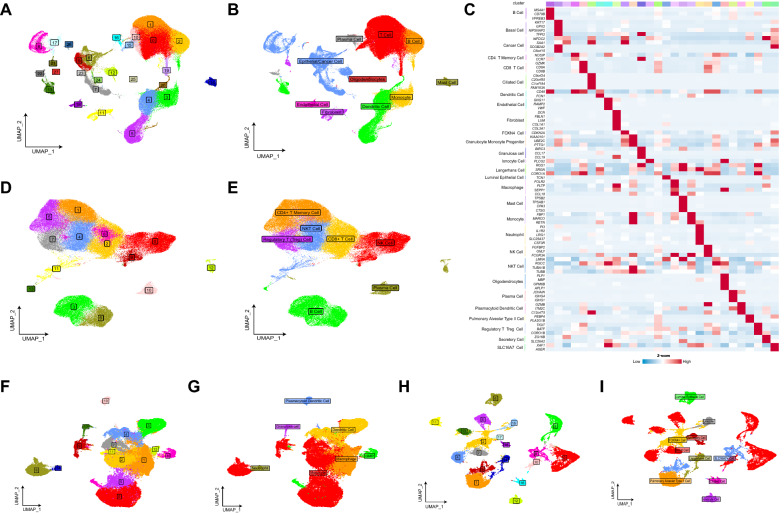
Single-cell analysis: the cell clusters and their Marker were obtained by reduced-dimensional and clustering. Twenty-eight cell clusters (**A**) were obtained after the first level classification, and ten cell types (**B**) were identified by marker gene annotation. Fourteen cell clusters (**D**) were obtained after the second-level classification of lymphoid immune cells, and seven cell types (**E**) were identified by marker gene annotation. Fifteen cell clusters (**F**) were obtained after the second-level classification of myeloid immune cells, and seven cell types (**G**) were identified by marker gene annotation. Eighteen cell clusters (**H**) were obtained from normal epithelial cells after secondary classification, and then nine cell types (**I**) were identified by marker gene annotation. (**C**) Heatmap of the expression level of Marker genes from twenty-eight cell types

All cells were clustered into 28 clusters by standard procedure and further annotated into ten cell types: T Cell, B Cell, Plasma Cell, Mast Cell, Monocyte, Dendritic Cell, Fibroblast, Endothelial Cell, Epithelial/Cancer Cell, Oligodendrocytes (Fig. 1A and B). Next, we performed further subgroup clustering on lymphoid immune cell clusters (T Cell, B Cell, Plasma Cell), myeloid immune cell clusters (Monocyte, Dendritic Cell), and Epithelial/Cancer Cell respectively.

The lymphoid immune cell cluster is further divided into 14 clusters. Seven main subgroups are identified by annotation: Natural Killer (NK) Cell, CD4 + T Memory Cell, CD8 + T Cell, B Cell, Natural Killer T (NKT) Cell, Regulatory T (Treg) Cell, and Plasma Cell (Fig. 1D and E). Subsequently, myeloid immune cells were roughly further divided into 15 cell clusters. The annotation identified seven major subgroups: Monocyte, Macrophage, Dendritic Cell, Granulocyte-Monocyte Progenitor (GMP), Plasmacytoid Dendritic Cell, Granulosa Cell, and Neutrophil (Fig. 1F and G). For Epithelial/Cancer Cell, we used the CopyKAT algorithm to distinguish between normal epithelial cells and cancer cells (Additional file 2: Fig. S2B). We then further analyzed normal epithelial cells to obtain 18 cell clusters. The annotation identified nine main subgroups: Basal Cell, Pulmonary Alveolar Type II Cell, FOXN4 + Cell, Luminal Epithelial Cell, SLC16A7 + Cell, Ionocyte Cell, Langerhans Cell, Ciliated Cell, and Secretory Cell (Fig. 1H and I). Marker gene expression levels for 28 cell types were shown in Fig. 1C, indicating that different cell types had their own specific marker genes. Cell annotation information is shown in the Additional file 5: Table S1.

### Communication network research in the TIME

**Fig. 2:**
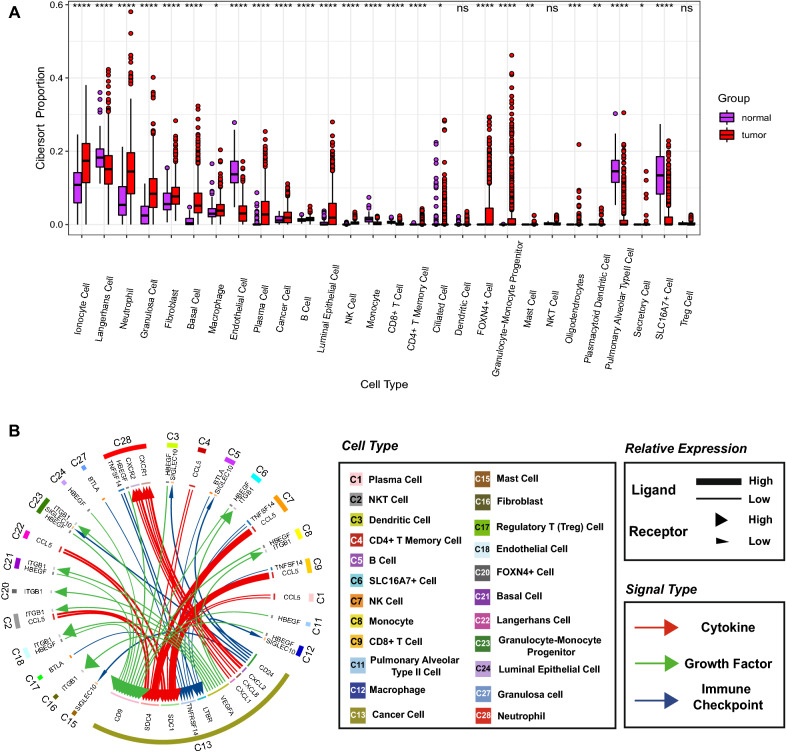
The abundance of 28 cell types in Bulk RNA-seq and cell interaction network in scRNA-seq. **A** Twenty-eight cell types were annotated to the TCGA queue by CIBERSORT. (*P < 0.05; **P < 0.01; ***P < 0.001; ****P < 0.0001). **B** The cellular communication network in which Cancer cells interact with other cells

We then annotated the above 28 cells in the TCGA cohort, displayed the proportion of cellular abundance (Additional file 2: Fig. S2C), and found that most of the cells were significantly different between normal and tumour patients (Fig. 2A). It is interesting to note that Neutrophil content was higher in patients, especially in tumor patients, and was significantly higher than in regular patients, which may be related to the tumor promoting and tumor suppressing properties of Neutrophils in tumors.

Subsequently, the cellular interaction network in the microenvironment was investigated based on 28 cell clusters (Fig. 2B). Concerning immune checkpoints, highly expressed *TNFSF14* in Neutrophils, NK Cells, and Monocytes, *BTLA* in Treg Cells, Granulosa Cells, and B Cells, together with *LTBR* and *TNFRSF14* in Cancer Cells, interact to help kill Cancer Cells. In addition, *CD24*, which is highly expressed in Cancer Cells, synergizes with *SIGLEC10* in Dendritic Cells, Macrophages, GMP Cells, B Cells, and Mast Cells to mediate tumor immune escape. For cytokines, *CCL5* is highly expressed in CD8 + T cells, NK Cells, NKT Cells, CD4 + T Memory Cells, Plasma Cells, and Langerhans Cells and strongly interacts with *SDC1* and *SDC4* in Cancer Cells, affecting cancer progression, development, and the survival. Chemokines *CXCL1*, *CXCL2*, and *CXCL8* are highly expressed in Cancer Cells and act on *CXCR1* and *CXCR2*, which are highly expressed in Neutrophil chemotactic the activity of Neutrophils and promote the generation of tumor immune microenvironment. Regarding growth factors, *HBEGF* in Monocytes, Neutrophils, Dendritic Cells, GMP Cells, Macrophages, Alveolar type II Cells, SLC16A7 + Cells, and Endothelial Cells, Basal Cells, and Luminal epithelial Cells was associated with high levels in Cancer Cells expressed *CD9* interacts to mediate tumorigenesis and proliferation. We also found that Cancer Cells interact with *ITGB1* expressed in Fibroblasts, Endothelial Cells, SLC16A7 + Cells, FOXN4 + Cells, Basal Cells, GMP Cells, Monocytes, and NKT Cells through angiogenic signal molecules (*VEGFA*), which may stimulate tumor growth and metastasis.

### Different differentiation characteristics of neutrophils

**Fig. 3:**
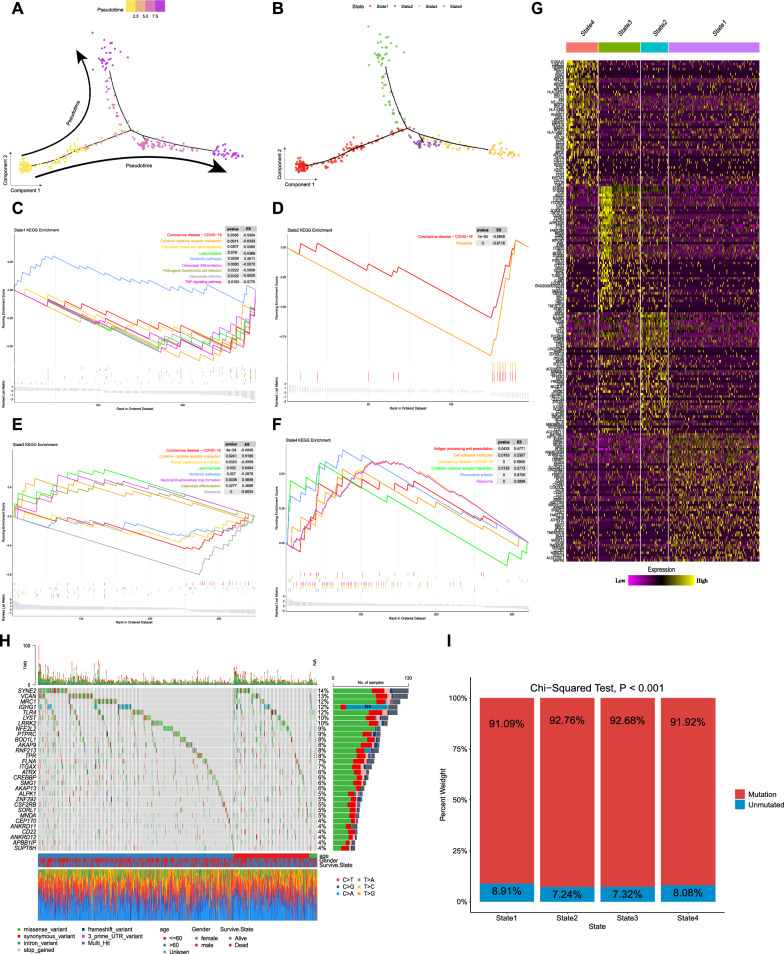
Pseudotime analysis of Neutrophils and mutational analysis of NDRGs. According to the pseudotime (**A**) of Neutrophils, the cell population was divided into four different differentiation states (**B**), and NDRGs (**G**) were obtained by difference analysis of differentiation states. GSVA (KEGG terms) analyzes four different differentiation states (**C**–**F**). Top 30% mutation frequency of NDRGs and mutation type (**H**) and mutation status of NDRGs in different states (**I**)

Next, we used Monocle for pseudotime trajectory analysis of Neutrophil subsets. The results showed that Neutrophils were divided into four different differentiation states (Fig. 3A and B). In the NDRGs of mutation frequency top 30% (Fig. 3 H), seven genes with mutation rate ≥ 10% were found, and the mutation rates of NDRGs in different differentiation states were all over 91% (Fig. 3I and Additional file 3: Fig. S3D). The above results demonstrate that NDRGs are highly mutated and heterogeneous, suggesting that NDRGs play a critical role in Neutrophils influencing tumorigenesis and development. Subsequently, GSEA was performed on the four states (Fig. 3C–F and Additional file 3: Fig. S3A–C). It was found that state one was significantly up-regulated in Metabolic and showed a down-regulation trend in the TNF signaling pathway and regulation of apoptotic signaling pathway, indicating that state one is mainly differentiated related to the initial state and participates in the occurrence and development of tumors, showing a tumor-promoting effect. State two was down-regulated in multiple metabolic processes, including peptide biosynthetic process, and was highly down-regulated in Ribosome-related pathways, indicating that state two is a new state with complete differentiation and down-regulation of metabolism. State three is similar to state two, but state three is significantly up-regulated in Neutrophil extracellular trap formation, Neutrophil chemotaxis, and GTPase activity, suggesting that state three is involved in Neutrophil chemotaxis. Prominently, state four is significantly up-regulated in Antigen processing and presentation, positive regulation of leukocyte activation, and signaling receptor regulator activity. Compared with the other states, the activity of Coronavirus disease—COVID-19 was increased in state four, indicating that state four is a state that produces immunoreactive activity, which manifests as an immune antitumor effect. Overall, we found four distinct states of Neutrophil differentiation and their NDRGs of high mutagenicity and heterogeneity.

### Establish a stable and effective prognostic risk model

**Fig. 4:**
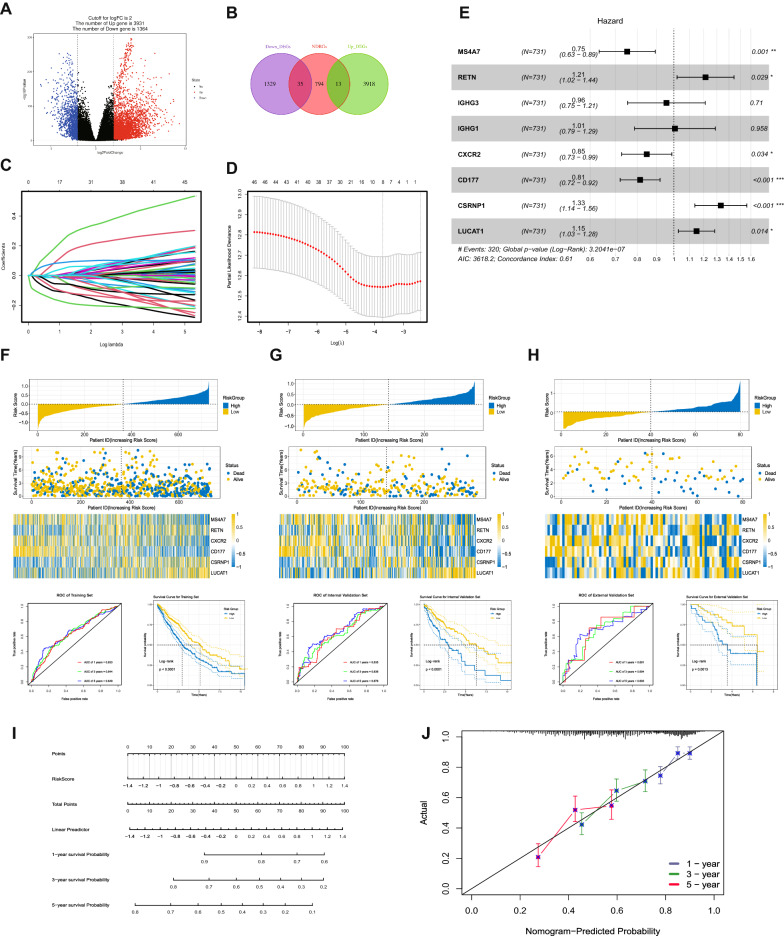
Construction and verification of the prognostic risk model. The intersection (**B**) of the differential gene (**A**) and NDRGs. Eight NDRGs with prognostic characteristics were screened by the Elastic Net Regression algorithm (**C**, **D**), and six prognostic risk model genes were confirmed by Multivariate Cox (**E**). The risk score distribution, patient status, mRNA expression heatmap, ROC curve, and KM survival curve of the training sets (**F**), the internal validation set (**G**), and the external validation sets (**H**). (**I**) Nomogram of the prognostic risk model. (**J**) The nomogram calibration curves to predict the 1-, 3-, and 5-year survival

To construct a prognostic risk model, the TCGA cohort was split into the training sets (n = 731) and the internal validation sets (n = 283), and the GEO cohort (n = 80) for external validation sets. In addition, NDRGs and DEGs (Fig. 4A) were intersected (Fig. 4B), and the intersected genes were used to build the prognostic risk model. Firstly, we used the rate of intersection genes/housekeeping genes (*ACTB, GAPDH, TFRC, TUBB*) to establish the prognostic risk model so that the results obtained can be more widely used. Then, using the Elastic Net Regression algorithm, eight critical genes related to prognosis were identified (Fig. 4C and D). Finally, six stable essential prognostic genes (Fig. 4E) and their regression coefficients were identified by Multivariate Cox Regression, and the final prognostic model was: Risk Score = 0.193*RETN_*Exp*_-0.285*MS4A7_*Exp*_-0.165*CXCR2_*Exp*_-0.206*CD177_*Exp*_ + 0.287*CSRNP1_*Exp*_ + 0.138*LUCAT1_*Exp*_.

The time-dependent ROC curve was used to evaluate the prognostic ability of the risk scoring model, and the 1-year, 3-year, and 5-year AUCs of the training set, internal validation set, and external validation set were all greater than 0.6, indicating that the prognostic risk model has a strong predictor for the survival of NSCLC patients. Kaplan–Meier survival curves showed that survival rates were significantly different among the three cohorts grouped by risk score (Log-rank, *P* < 0.0001), showing that the risk score could be used as a predictor of patient prognosis. (Fig. 4F–H). In addition, we established a nomogram using the prognostic signature (Fig. 4I). The calibration curves for the 1-year, 3-year, and 5-year survival indicate a high degree of overlap between the actual survival rate and the survival rate predicted by the nomogram (Fig. 4J). This suggests that the nomogram has an excellent predictive value.

### Immune prediction and clinical application of prognostic risk model

**Fig. 5:**
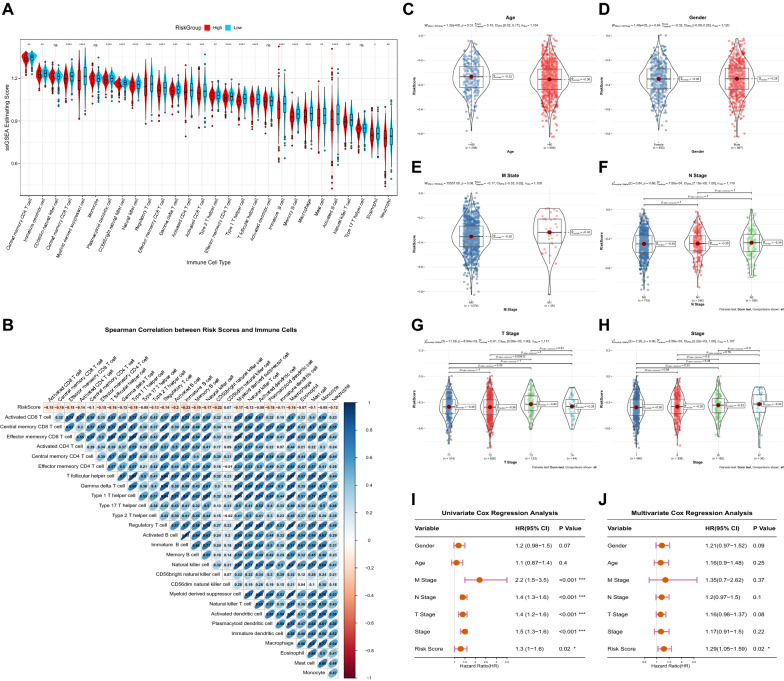
Immune predictive performance and clinical predictive power of the prognostic model. **A** After grouping the risk score according to the median, check the abundance of 28 immune cells in the high-risk and low-risk groups. **B** Spearman correlation analysis between risk score and the abundance of 28 kinds of immune cells. **C**–**H** Age, Gender, M stage, N stage, T stage, and Stage distribution of the patients in the high-risk and low-risk groups. Univariate Cox Regression (**I**) and Multivariate Cox Regression (**J**) analysis of clinical information of TCGA cohorts. (*P < 0.05; **P < 0.01; ***P < 0.001; ****P < 0.0001)

The ssGSEA result found that the content of most immune cells in the high-risk group was significantly lower than that in the low-risk group, indicating that there were more immune components in the tumor microenvironment of the low-risk group and also that the immune prognosis of the high-risk group was worse (Fig. 5A). As the risk score increases, immune cell composition decreases, and the effect of immunotherapy worsens. The above results showed that the prognostic risk model was involved in regulating the immune microenvironment and can be used as an indicator to predict the efficacy of immunotherapy.

After confirming the performance of six prognostic genes in predicting immune response in patients with NSCLC, we investigated the relationship between clinical characteristics and risk score. There was a significant statistical difference between Age and T stage (Fig. 5C–H), showing that risk score was related to Age and T stage. Next, we explored the clinical application of the six prognostic gene models in predicting patient outcomes using Univariate Cox Regression and Multivariate Cox Regression analyses. Univariate Cox results showed that risk score was significantly associated with prognosis (*P* = 0.02, HR = 1.3, 95% CI 1–1.6) (Fig. 5I), and Multivariate Cox results also proved that risk score was an independent prognostic factor for NSCLC (*P* = 0.02, HR = 1.29, 95% CI 1.05–1.59) (Fig. 5J). These results confirmed that the six prognostic gene risk model has perfect prognostic efficiency.

### Explore the functional of six prognostic genes

**Fig. 6:**
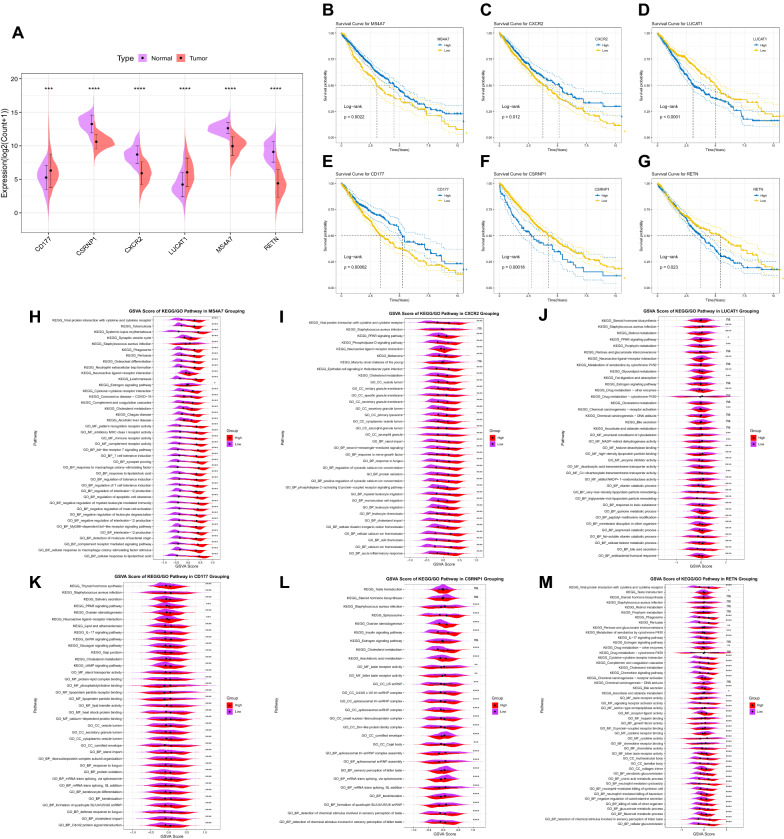
Expression levels, survival analysis and functional studies of six prognostic genes in the TCGA cohort. **A** Expression levels of six prognostic genes in the TCGA cohort. **B**–**G** KM survival curves of six prognostic genes in the TCGA cohort. After grouping *MS4A7* (**H**), *CXCR2* (**I**), *LUCAT1* (**J**), *CD177* (**K**), *CSRNP1* (**L**) and *RETN* (**M**) at high and low levels, the enriched KEGG and GO pathways were scored for GSVA

Next, we further explored the six prognostic genes’ expression, survival, and pathway alterations. Firstly, for *MS4A7*, it was down-regulated in tumors, and the Kaplan–Meier survival curves indicated that low expression of *MS4A7* predicts a worse prognosis (Fig. 6B). The results of GSVA after low expression showed that the activities of various immune response pathways, including Immune Receptor activity were down-regulated, indicating that *MS4A7* was involved in various immune and anti-inflammatory responses (Fig. 6H). Similarly, *CXCR2* is expressed at a low level in tumor tissues (Fig. 6A), and the prognosis of *CXCR2* with low expression is worse (Fig. 6D). After the low expression of *CXCR2*, it was found that the immune activity-related pathways such as Cytokine and Cellular Calcium Ion Homeostasis also showed a down-regulated state (Fig. 6I), indicating that tumor down-regulated some immune stress responses by decreasing the expression of *CXCR2* to ensure that it was not killed by immune cells. As for *LUCAT1*, it is involved in various processes promoting the occurrence and development of NSCLC, which was fully illustrated by the evidence of its high expression level in tumors and worse prognosis after high expression (Fig. 6A and C). Furthermore, after the overexpression of *LUCAT1*, most biological Metabolic pathways, including Glycerolipid metabolism and cancer-related pathways such as the Chemical carcinogenesis−receptor activity pathway, increased significantly (Fig. 6J), which also proved the role of *LUCAT1* in promoting cancer.

On the contrary, the *CD177* gene is highly expressed in tumor tissues (Fig. 6A). However, the prognosis was relatively better after its high expression (Fig. 6G), which may be related to the activation of Neutrophils promoted by *CD177*, and the content of neutrophils is higher in tumor tissue. After overexpression, it was found that the activities of signal pathways such as IL-17 signaling pathway were significantly up-regulated (Fig. 6K), and *CD177* was positively correlated with the abundance of most immune cells (Additional file 4: Fig. S4E), suggesting that *CD177* plays an immune effect of chemotactic neutrophils in NSCLC. Furthermore, *CSRNP1* and *RETN* were down-regulated in tumor tissues (Fig. 6A), but the down-regulation of both predicted a better prognosis (Fig. 6E and F). The results of Spearman correlation analysis showed that they were positively correlated with the abundance of most immune cells (Additional file 4: Fig. S4D and F). After low expression of *CSRNP1*, it was found that the activity of many transcription-related pathways, including spliceosomal snRNP assembly, was decreased (Fig. 6L), speculated that *CSRNP1* was involved in the growth and differentiation of cells. After low expression of *RETN*, it was found that the activities of the Chemokine signaling pathway and Neutrophil related pathways were significantly down-regulated (Fig. 6M), suggesting that *RETN* was related to multiple immune stress responses.

### Small molecular compounds docking of prognostic genes

**Fig. 7:**
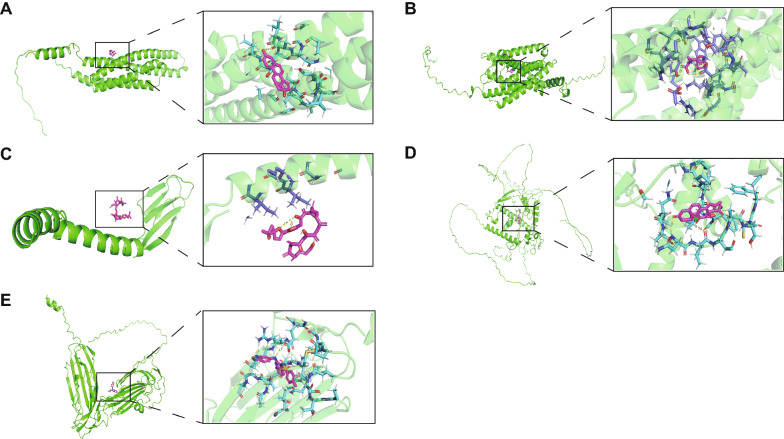
The docking results of proteins encoded by prognostic genes with small molecular compounds. The docking results of *MS4A7* with Estradiol (**A**). The docking results of *CXCR2* with Abrine (**B**). The docking results of *RETN* with Ionomycin (**C**). The docking results of *CSRNP1* with Beclomethasone (**D**). The docking results of *CD177* with XL147 (**E**)

In this study, we used screening of the CTD database, Autodock molecular docking, and drug toxicology studies to identify drugs targeted to prognostic genes. We found that Estradiol was able to bind tightly to *MS4A7* (Fig. 7A) and upregulate *MS4A7* mRNA expression, and their simulated binding energy for molecular docking was − 4.23 (kcal/mol). Estradiol, a naturally occurring endogenous circulating hormone in women, is often used in the treatment of conditions associated with estrogen depletion. Estradiol overdose can cause changes including red number of red blood cells and uterine weight. The results of the molecular docking analysis indicated that among the small molecule compounds that ameliorated the increase in mRNA expression of *CXCR2*, Abrine stood out with an optimal docking binding energy of − 4.58 (kcal/mol) (Fig. 7B). Abrine, also known as N (alpha)-methyl-L-tryptophan, is an N (alpha)-methyl derivative of L-tryptophan, which effectively reduces the breakdown activity of tryptophan and improves the efficacy of immunotherapies. At this time, there are no details of the toxic effects other than the lethal dose reports. Ionomycin can efficiently bind *RETN* and increase its level of mRNA expression (Fig. 7C), their docking energies being − 7.91 (kcal/mol). Ionomycin is a calcium ion transporter with antitumor activity produced by Streptomyces polymerases, which may increase the intracellular calcium ion level and ultimately result in apoptosis. No toxicity has been reported for Ionomycin, except for lethal dose reports. Interestingly, Beclomethasone exhibited a high level of docking binding energy of up to − 10.97 (kcal/mol) when searching for small-molecule compounds that enhance the expression of *CSRNP1* (Fig. 7D). Beclomethasone is a prototypical glucocorticoid receptor agonist that functions as an anti-inflammatory as well as an anti-asthma. No details of the toxic effects were reported except for the value of the lethal dose. Finally, in screening for small molecule compounds that upregulate *CD177* mRNA, XL147 distinguished itself by its − 8.36 (kcal/mol) molecular docking binding energy (Fig. 7E). The combination of XL147 and N-nitroso-tris-chloroethylurea resulted in increased gene expression of *CD177*. XL147, a sulfonamide, is a selective PI3K inhibitor for cancer treatment. More than or equal to 0.1% composition of XL147 was certified by the International Agency for Research on Cancer as a non-human carcinogen. In summary, we have selected five small molecular compounds that are conducive to improving the worse prognosis caused by five prognostic genes, providing a new research idea for targeted therapy of NSCLC.

## Discussion

Lung cancer is the most widespread cancer globally, and NSCLC is the primary subtype of lung cancer. Most patients are resistant to immunotherapy, which may be related to their TIME. With the rapid development of scRNA-seq in cancer medicine, it is now possible to study highly heterogeneous tumors, including NSCLC, which will bring epochal shifts in the understanding of TIME and the exploration of novel cellular biomarkers [25, 26].

This study comprehensively analyzes TIME in NSCLC by integrating two large scRNA-seq datasets. After quality control and dimensionality reduction clustering, ten cell types were initially annotated, and further subdivisions resulted in 28 major cell types. Annotation of cellular abundance in patients in the TCGA cohort based on the overall expression of mRNAs characteristic of 28 cells found that most cells differed significantly between tumors and normal tissues. Neutrophils were more abundant in tumor tissue, consistent with previous studies [27]. Tumor-immune cell interactions lead to metabolic competition within the tumor ecosystem, which limits the effective supply of nutrition, and thereby hinders immune cell function. It has been reported that IL-18 may positively regulate autophagy to promote myocardial cell mitochondrial function and the steady state maintenance of gap junctional turnover [28]. Close binding between mitochondria and gap junctions regulates the ionic permeability of gap junctions and influences metabolic reprogramming [29, 30]. Multiple reliable ligand-receptor pairs were collected using cellular communication analysis in the research, characterizing the complex regulatory network in the NSCLC tumor microenvironment. The immune checkpoint *TNFSF14* in Neutrophils, NK Cells, and Monocytes and *BTLA* in Treg Cells, Granulosa Cells, and B Cells interact with *LTBR* and *TNFRSF14* in Cancer Cells to mediate cytotoxicity and promote tumor killing [31–34]. In addition, the high expression of *CD24* in Cancer Cells affects the expression of *SIGLEC10* in Dendritic Cells, Macrophages, GMP Cells, B Cells, and Mast Cells, which in turn affects immune disorders and leads to tumor immune escape responses [35]. The highly expressed cytokine *CCL5* in CD8 + T Cells, NK Cells, NKT Cells, CD4 + T Memory Cells, Plasma Cells, and Langerhans Cells work together with *SDC1* and *SDC4* in Cancer Cells to affect the occurrence, development, and mediation of cancer survival of cancer cells [36]. Cytokines *CXCL1*, *CXCL2*, and *CXCL8* in Cancer Cells interact with *CXCR1* and *CXCR2* in Neutrophils, chemotactic the activity of Neutrophils, and promote the generation of tumor immune microenvironment [37]. The growth factor *HBEGF*, which is highly expressed in Monocytes, Neutrophils, Dendritic Cells, GMP Cells, Macrophages, and various Epithelial Cells, interacts with *CD9* expressed in Cancer Cells to help mediate tumorigenesis and proliferation [38]. We also found that Cancer Cells interact with *ITGB1*, highly expressed in multiple cells such as Fibroblasts, Endothelial Cells, and Basal Cells, through angiogenesis signal molecules (*VEGFA*) to stimulate tumor growth and metastasis [39]. This discovery provides a new research idea for tumor immunotherapy. Further mining the high heterogeneity of Neutrophils, we identified Neutrophil states with four distinct differentiation fates through developmental trajectory analysis. Using GSEA to functionally characterize signatures of differentiation, we found that this pattern of differentiation is intrinsically linked to intratumoral immune and metabolic biology as well. NDRGs in different differentiation states showed a highly mutated state, with a mutation rate greater than 91%, indicating that NDRGs play a crucial role in the occurrence and development of tumors. Based on the above findings, we established a prognostic risk model consisting of six NDRGs, *MS4A7, CXCR2, CSRNP1, RETN, CD177*, and *LUCAT1,* according to the rate of four reference genes (*ACTB, GAPDH, TFRC, TUBB*). Overall, the model was suitable for various detection data and can effectively predict the prognosis and immunotherapy response of NSCLC patients, providing a theoretical basis for formulating individualized treatment for patients.

Studies have shown that *MS4A7* has a particular prognostic value in ovarian cancer [40] and glioma [41]. Our research found that *MS4A7* mainly mediates most immune-related pathways, such as immune receptor activity, but *MS4A7* is down-regulated in tumor tissues, and its low expression levels indicate a worse prognosis. It is speculated that the tumor produces immune tolerance by down-regulating the expression of *MS4A7*, leading to a worse prognosis. As a LncRNA, *LUCAT1* is involved in the occurrence and development of lung cancer. Studies have found that *LUCAT1* can promote the metastasis of lung adenocarcinoma cells and glycolysis by regulating the miR-4316/VEGFA axis [42]. It has been found in this research that the overexpression of *LUCAT1* increases the activity of most metabolic and carcinogenic pathways, and *LUCAT1* has a significant negative correlation with immune cells. It indicated that *LUCAT1* was an oncogene that promoted the occurrence and development of tumors by affecting metabolic pathways in the tumor microenvironment and inhibiting the immune response of immune cells, resulting in a lousy prognosis. Recent studies have shown that *CXCR2* can be used as a valuable independent prognostic marker in patients with cholangiocarcinoma, and its mediated immune response may have a tumor inhibition effect on cholangiocarcinoma cells [43]. Our study confirmed that the down-regulation of *CXCR2* is associated with a poor prognosis. *CXCR2* has a significant positive correlation with most immune cells, and the activity of most immune response pathways, including acute inflammatory reactions, is increased, suggesting that *CXCR2* can inhibit cancer by inducing immune responses, and significant down-regulation of tumor tissues is also the main reason for worse prognosis. The tumor occurrence is usually related to the inflammatory reaction caused by excessive adipose tissue. It has been reported that the fat factor *RETN* can activate obesity-related inflammatory responses through the combined action of the pro-inflammatory cytokine IL-1β [44]. Studies have found that high expression of *RETN* predicts a adverse prognosis because *RETN* promotes an inflammatory response. Interestingly, *RETN* is low expressed in the tumor. After the simulation of down-regulation, it was found that the activities of immune cell chemotactic related pathways were decreased, and a positive correlation between *RETN* and immune cells. It indicated that *RETN* helped improve the chemotaxis of immune cells, and tumors could ensure their survival by down-regulating *RETN*. It has been reported that the *CSRNP1* gene can be used as a prognostic biomarker [45, 46] for many cancers, indicating the essential prognostic value of *CSRNP1*. When *CSRNP1* is simulated to be down-regulated, the activities of various biological modification-related pathways, including Spliceosome activity, are down-regulated. In addition, *CSRNP1* was positively correlated with most immune cells. It is speculated that *CSRNP1* is involved in the growth and development of immune cells, and the tumor produces immune resistance by down-regulating *CSRNP1*. However, because *CSRNP1* is also involved in the growth and modification of tumor cells, its high level of expression will lead to a dreadful prognosis and high-risk score. As a Neutrophil surface glycoprotein, *CD177* triggers Neutrophil degranulation and superoxide production. Recently reported, *CD177* can regulate *PDPN* and thus affect the physiological changes of cancer-related fibroblasts, which seems to be a new therapeutic target [47]. *CD177* was up-regulated in tumor tissue and correlated with Neutrophil content in this study. When *CD177* is overexpressed, many immune response pathways and biological regulatory pathways are significantly up-regulated, such as the IL-17 signaling pathway and the protein oxidation pathway. Therefore, low expression of *CD177* indicates a decrease in the content of immunocytes, especially Neutrophils, and a corresponding decrease in antitumor activity, resulting in a worse prognosis. Understanding the function of prognostic genes and their causes of dreadful prognosis will help to propose targeted therapy options.

Today, reorientation of drug function is a novel strategy for disease treatment. As disease mechanisms continue to deepen and treatment plans continue to be refined, a variety of drugs for treating disease including Valproic acid [48] for the treatment of epilepsy have been applied to the treatment of cancer. Therefore, based on this strategy, we conducted targeted drug screening of prognostic genes with a view to proposing a therapeutic approach that modulates poor prognosis. As a small molecule compound that can efficiently bind to and upregulate *MS4A7* expression, more than 95% of estradiol in the bloodstream binds to sex hormone-binding globulin (SHBG) and alumina, which is commonly used to treat diseases related to estrogen reduction. However, excessive intake of estrogen can result in side effects such as nausea, vomiting, and vein thrombosis. Furthermore, estradiol functions as an immunomodulator in immune and inflammatory processes [49]. Abrine also showed an exceptional performance in increasing *CXCR2* expression. Abrine was shown to be a competitive inhibitor of indoleamine-2,3-dioxygenase (IDO) in in vitro experiments, which could effectively reduce tryptophan degradation activity and enhance the efficacy of immunotherapies. Abrine is currently being used in conjunction with a series of chemotherapeutic drugs such as cisplatin, doxorubicin and paclitaxel, and has been shown to have excellent synergistic effects [50]. Zhang et al. has shown that Abrine can regulate hepatocellular carcinoma cell growth and apoptosis via the KAT5/PD-L1 axis [51]. The natural product ionomycin used in this study had high affinity to *RETN*. The natural product of Ionomycin, which is found in Streptomyces polymerases, is also a calcium transporter that can increase the intracellular calcium level, which is linked to the activation of the endonuclease in lymphocytes and the reduction in the ratio of Bcl-2 to Bax, ultimately mediating apoptosis [52, 53]. Beclomethasone was one of the compounds with up-regulation of *CSRNP1* that exhibited high affinity docking binding energy. Beclomethasone is a Corticosteroid with anti-inflammatory and immunomodulating properties for chronic obstructive pulmonary disease and COVID-19. It has been reported that Beclomethasone inhibits normal physiologic neutrophil migration and neutrophil chemotaxis upon detection of trauma induced inflammation [54, 55]. In the cohort screened for drugs that promoted increased *CD177* mRNA expression, XL147 was found to have high affinity for the *CD177* mRNA. XL147 is a potent inhibitor of oral bioavailability and a member of the class I PI3K family of lipid kinases. In a variety of clinical cancer models, XL147 treatment has been found to significantly inhibit PI3K pathway signaling in tumors and lead to significant inhibition of tumor growth or tumor shrinkage [56, 57]. Based on the data from the five targeted drugs targeting the five aforementioned prognostic genes, our study has proposed a novel targeted therapy scheme consisting of a combination of multiple drugs, which will help improve the poor prognosis brought by the five prognostic genes and improve patient survival rate. Among various targeted therapeutics, there has also been increased interest in novel biomaterials, including nanomaterials [58] and hydrogel materials for hyaluronic acid [59]. As a novel antioxidant with low toxicity and high efficacy, the nano-antioxidant is superior to the traditional antioxidant in improving superoxide dismutase and catalase activities in organisms, and has a lower biological toxicity [60]. Hyaluronic acid-constructed hydrogel materials are brand new drug delivery vehicles, which can effectively reduce cytotoxicity, deliver drugs safely and efficiently to the site of action, and allow drugs to play the largest role. Of the five gene-targeted drugs chosen in this study, the primary goal is to regulate mRNA expression of prognostic genes. However, further research is needed on how to deliver drugs to drug targets. The drug delivery scaffold built with novel biomaterials may be an excellent choice.

The novelty of this study lies in the integration of large scale scRNA-seq to analyze the NSCLC regulatory network and further resolve the complex interactions within the TIME. We also employ a novel strategy of combining the Elastic Net Regression algorithm with housekeeping genes ratios for prognostic risk modeling. Further investigation discussing prognostic gene function and drug targeting research is also novel in this study. At the same time, there remain limitations to this study. First, although we had performed a batch correction for the two scRNA-seq data, the essential batch effect still exists. In that regard, future integration studies could begin with sequenced documents to ensure consistency and accuracy of data. Secondly, our results are still in the analytical and speculative stage and have not been experimentally validated, which is what future work will need. The combined therapeutic value of these five targeted drugs at the cellular and animal level will be the subject of future work. Furthermore, on the basis of our prognostic risk model, we hope to establish a shared network platform to aid in clinical diagnosis and prognostic therapy in NSCLC.

## Conclusion

In this research, we combine two large-scale scRNA-seq data to illustrate the complex cellular communication network in TIME and characterize four differentiation states and NDRGs of Neutrophils. We were able to establish a prognostic risk model that could be used to predict patient prognostic performance and immunotherapeutic efficacy. Lastly, causes of adverse prognosis caused by prognostic genes were discussed, and drugs were screened for the presence of prognostic genes, leading to new insights for targeted therapy.

## Supplementary Information

**Additional file 1: Figure S1.** Research Workflow.

**Additional file 2: Figure S2.** Single-cell analysis and CIBERSORT analysis. (**A**) The patient cells were screened out after UMAP plotted quality control. (**B**) The CopyKAT algorithm distinguishes cancer cells and normal cells. (**C**) CIBERSORT counts distinct cell abundances in the TCGA cohort.

**Additional file 3: Figure S3.** GSEA analysis (GO terms) of the four differentiation states and mutation types of NDRGs. GSEA enrichment scores for Biological Process (**A**), Cellular Component (**B**), and Molecular Function (**C**) terms in four differentiation states. (**D**) Mutation types of NDRGs.

**Additional file 4: Figure S4.** Spearman correlation analysis of six prognostic genes and abundance of 28 immune cells. Spearman correlation analysis of *MS4A7*(**A**), *CXCR2*(**B**), *LUCAT1*(**C**), *CSRNP1*(**D**), *RETN*(**E**), *CD177*(**F**), and the abundance of 28 immune cells, respectively.

**Additional file 5: Table S1.** Cell cluster annotation information. (**a**) The First levels of dimensionality reduction and clustering. The Second levels of dimensionality reduction and clustering: lymphoid immune cells (**b**), myeloid immune cells (**c**), and normal epithelial cells (**d**).

## Data Availability

The datasets generated analysed during the current study are available in the GEO and TCGA repository, including GSE131907, GSE148071, GSE81089, TCGA-LUAD and TCGA-LUSC.

## Abbreviations

NSCLC: Non-small cell lung cancer
TIME: Immune microenvironment
scRNA-seq: Single-cell RNA sequencing
NDRGs: Neutrophil differentiation-related genes
GEO: Gene Expression Omnibus Database
TCGA: The Cancer Genome Atlas
LUAD: Lung adenocarcinoma
LUSC: Lung squamous cell carcinoma
GSEA: Gene Set Enrichment Analysis
ROC: Receiver operating characteristic curve
ssGSEA: Single sample Gene Set Enrichment Analysis
GSVA: Gene set variation analysis
CTD: Comparative Toxicogenomics Database
NK Cell: Natural Killer Cell
NKT Cell: Natural Killer T Cell
Treg Cell: Regulatory T Cell
GMP Cell: Granulocyte-Monocyte Progenitor
