# Supplementary material for: Integrating Single-cell RNA-seq to construct a Neutrophil prognostic model for predicting immune responses in non-small cell lung cancer
Source: J Transl Med. 2022 Nov 18;20:531. doi: 10.1186/s12967-022-03723-x (PMC9673203; doi:10.1186/s12967-022-03723-x)
Supplement: Supplementary file 1 — Additional file 1: Figure S1. Research Workflow. [file 12967_2022_3723_MOESM1_ESM.pdf]

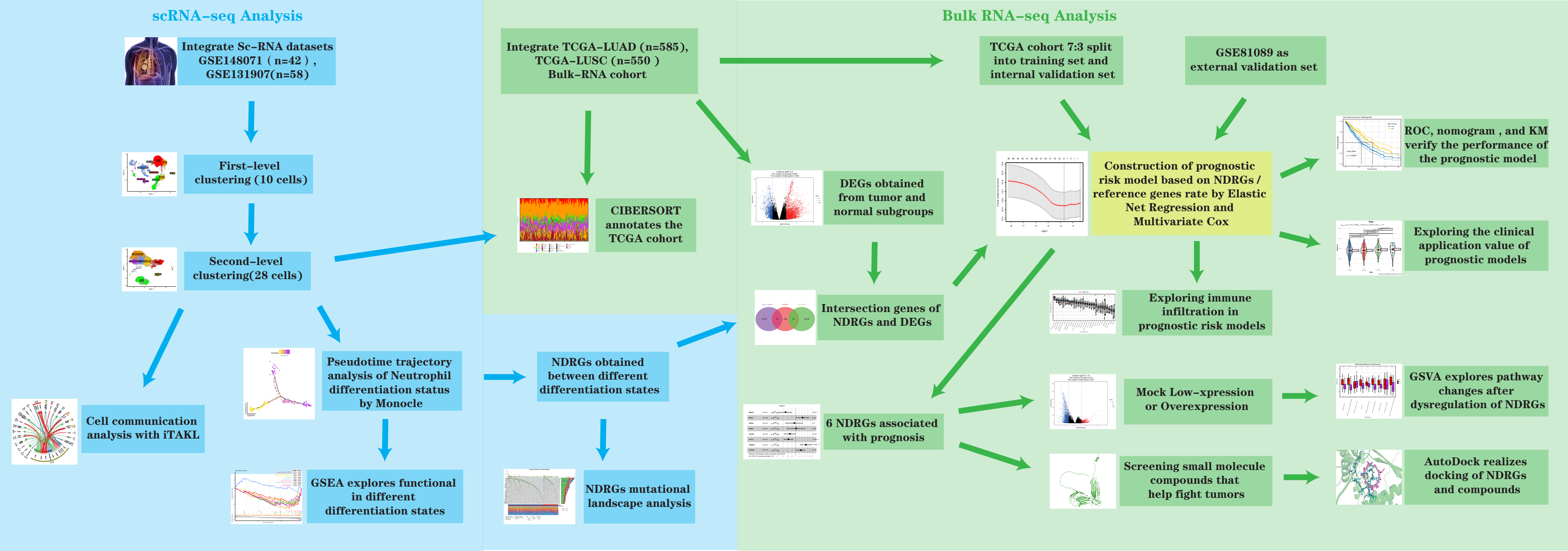
scRNA-seq Analysis
Bulk RNA-seq Analysis
Integrate Sc-RNA datasets GSE148071（n=42）, GSE131907(n=58)
First-level clustering (10 cells)
Second-level clustering(28 cells)
Cell communication analysis with iTAKL
Pseudotime trajectory analysis of Neutrophil differentiation status by Monocle
GSEA explores functional in different differentiation states
Integrate TCGA-LUAD (n=585), TCGA-LUSC (n=550) Bulk-RNA cohort
CIBERSORT annotates the TCGA cohort
NDRGs obtained between different differentiation states
NDRGs mutational landscape analysis
TCGA cohort 7:3 split into training set and internal validation set
GSE81089 as external validation set
DEGs obtained from tumor and normal subgroups
Intersection genes of NDRGs and DEGs
6 NDRGs associated with prognosis
Construction of prognostic risk model based on NDRGs / reference genes rate by Elastic Net Regression and Multivariate Cox
ROC, nomogram , and KM verify the performance of the prognostic model
Exploring the clinical application value of prognostic models
Exploring immune infiltration in prognostic risk models
Mock Low-xpression or Overexpression
GSVA explores pathway changes after dysregulation of NDRGs
Screening small molecule compounds that help fight tumors
AutoDock realizes docking of NDRGs and compounds
