# Supplementary figures and images for: Integrating Single-cell RNA-seq to construct a Neutrophil prognostic model for predicting immune responses in non-small cell lung cancer
Source: J Transl Med. 2022 Nov 18;20:531. doi: 10.1186/s12967-022-03723-x (PMC9673203; doi:10.1186/s12967-022-03723-x)

A

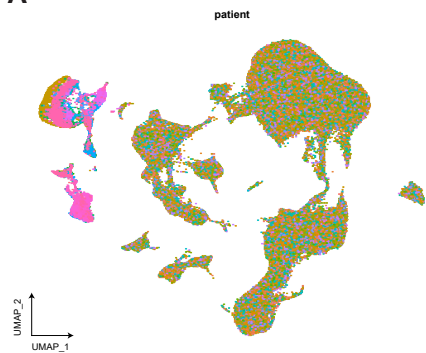

B

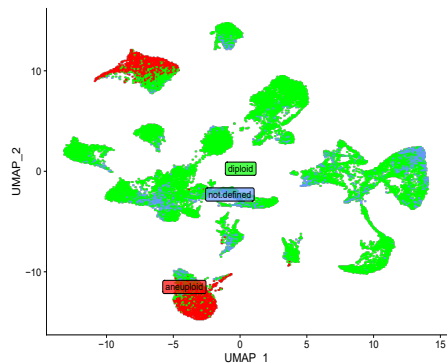

C

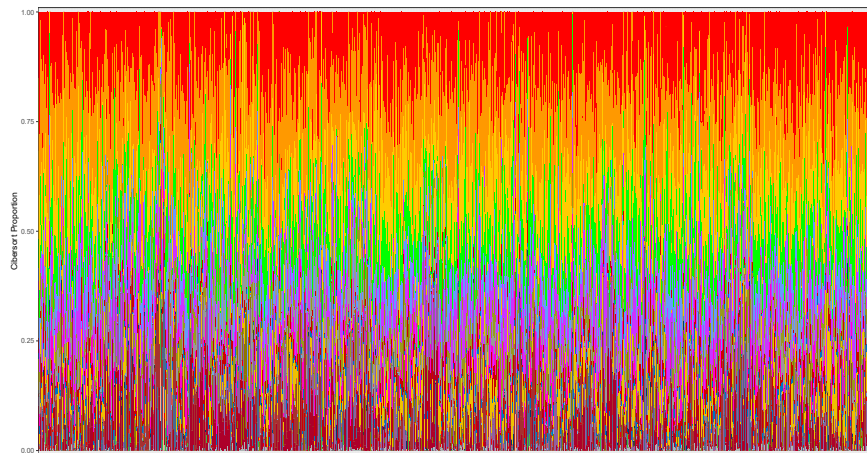

Supplement: Supplementary file 2 — Additional file 2: Figure S2. Single-cell analysis and CIBERSORT analysis. (A) The patient cells were screened out after UMAP plotted quality control. (B) The CopyKAT algorithm distinguishes cancer cells and normal cells. (C) CIBERSORT counts distinct cell abundances in the TCGA cohort. [file 12967_2022_3723_MOESM2_ESM.pdf]
