# Supplementary material for: Integrating Single-cell RNA-seq to construct a Neutrophil prognostic model for predicting immune responses in non-small cell lung cancer
Source: J Transl Med. 2022 Nov 18;20:531. doi: 10.1186/s12967-022-03723-x (PMC9673203; doi:10.1186/s12967-022-03723-x)
Supplement: Supplementary file 3 — Additional file 3: Figure S3. GSEA analysis (GO terms) of the four differentiation states and mutation types of NDRGs. GSEA enrichment scores for Biological Process (A), Cellular Component (B), and Molecular Function (C) terms in four differentiation states. (D) Mutation types of NDRGs. [file 12967_2022_3723_MOESM3_ESM.pdf]

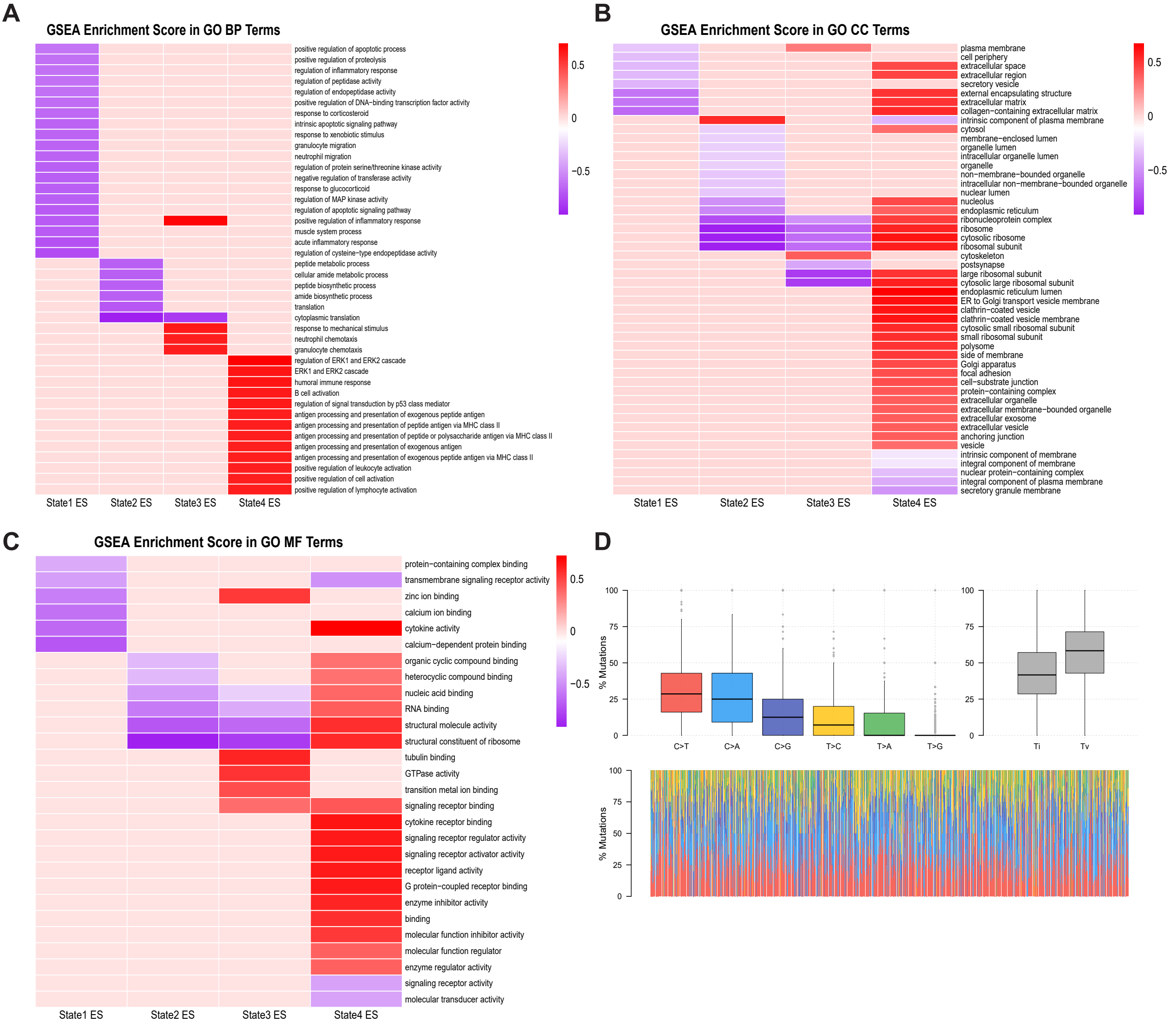
A
GSEA Enrichment Score in GO BP Terms
positive regulation of apoptotic process
positive regulation of proteolysis
regulation of inflammatory response
regulation of peptidase activity
regulation of endopeptidase activity
positive regulation of DNA−binding transcription factor activity
response to corticosteroid
intrinsic apoptotic signaling pathway
response to xenobiotic stimulus
granulocyte migration
neutrophil migration
regulation of protein serine/threonine kinase activity
negative regulation of transferase activity
response to glucocorticoid
regulation of MAP kinase activity
regulation of apoptotic signaling pathway
positive regulation of inflammatory response
muscle system process
acute inflammatory response
regulation of cysteine−type endopeptidase activity
peptide metabolic process
cellular amide metabolic process
peptide biosynthetic process
amide biosynthetic process
translation
cytoplasmic translation
response to mechanical stimulus
neutrophil chemotaxis
granulocyte chemotaxis
regulation of ERK1 and ERK2 cascade
ERK1 and ERK2 cascade
humoral immune response
B cell activation
regulation of signal transduction by p53 class mediator
antigen processing and presentation of exogenous peptide antigen
antigen processing and presentation of peptide antigen via MHC class II
antigen processing and presentation of peptide or polysaccharide antigen via MHC class II
antigen processing and presentation of exogenous antigen
antigen processing and presentation of exogenous peptide antigen via MHC class II
positive regulation of leukocyte activation
positive regulation of cell activation
positive regulation of lymphocyte activation
State1 ES
State2 ES
State3 ES
State4 ES
0.5
0
−0.5
B
GSEA Enrichment Score in GO CC Terms
plasma membrane
cell periphery
extracellular space
extracellular region
secretory vesicle
external encapsulating structure
extracellular matrix
collagen−containing extracellular matrix
intrinsic component of plasma membrane
cytosol
membrane−enclosed lumen
organelle lumen
intracellular organelle lumen
organelle
non−membrane−bounded organelle
intracellular non−membrane−bounded organelle
nuclear lumen
nucleolus
endoplasmic reticulum
ribonucleoprotein complex
ribosome
cytosolic ribosome
ribosomal subunit
cytoskeleton
postsynapse
large ribosomal subunit
cytosolic large ribosomal subunit
endoplasmic reticulum lumen
ER to Golgi transport vesicle membrane
clathrin−coated vesicle
clathrin−coated vesicle membrane
cytosolic small ribosomal subunit
small ribosomal subunit
polysome
side of membrane
Golgi apparatus
focal adhesion
cell−substrate junction
protein−containing complex
extracellular organelle
extracellular membrane−bounded organelle
extracellular exosome
extracellular vesicle
anchoring junction
vesicle
intrinsic component of membrane
integral component of membrane
nuclear protein−containing complex
integral component of plasma membrane
secretory granule membrane
State1 ES
State2 ES
State3 ES
State4 ES
0.5
0
−0.5
C
GSEA Enrichment Score in GO MF Terms
protein−containing complex binding
transmembrane signaling receptor activity
zinc ion binding
calcium ion binding
cytokine activity
calcium−dependent protein binding
organic cyclic compound binding
heterocyclic compound binding
nucleic acid binding
RNA binding
structural molecule activity
structural constituent of ribosome
tubulin binding
GTPase activity
transition metal ion binding
signaling receptor binding
cytokine receptor binding
signaling receptor regulator activity
signaling receptor activator activity
receptor ligand activity
G protein−coupled receptor binding
enzyme inhibitor activity
binding
molecular function inhibitor activity
molecular function regulator
enzyme regulator activity
signaling receptor activity
molecular transducer activity
State1 ES
State2 ES
State3 ES
State4 ES
0.5
0
−0.5
D
% Mutations
100
75
50
25
0
C>T
C>A
C>G
T>C
T>A
T>G
Ti
Tv
% Mutations
100
75
50
25
0
