# Supplementary material for: Integrating Single-cell RNA-seq to construct a Neutrophil prognostic model for predicting immune responses in non-small cell lung cancer
Source: J Transl Med. 2022 Nov 18;20:531. doi: 10.1186/s12967-022-03723-x (PMC9673203; doi:10.1186/s12967-022-03723-x)
Supplement: Supplementary file 4 — Additional file 4: Figure S4. Spearman correlation analysis of six prognostic genes and abundance of 28 immune cells. Spearman correlation analysis of MS4A7(A), CXCR2(B), LUCAT1(C), CSRNP1(D), RETN(E), CD177(F), and the abundance of 28 immune cells, respectively. [file 12967_2022_3723_MOESM4_ESM.pdf]

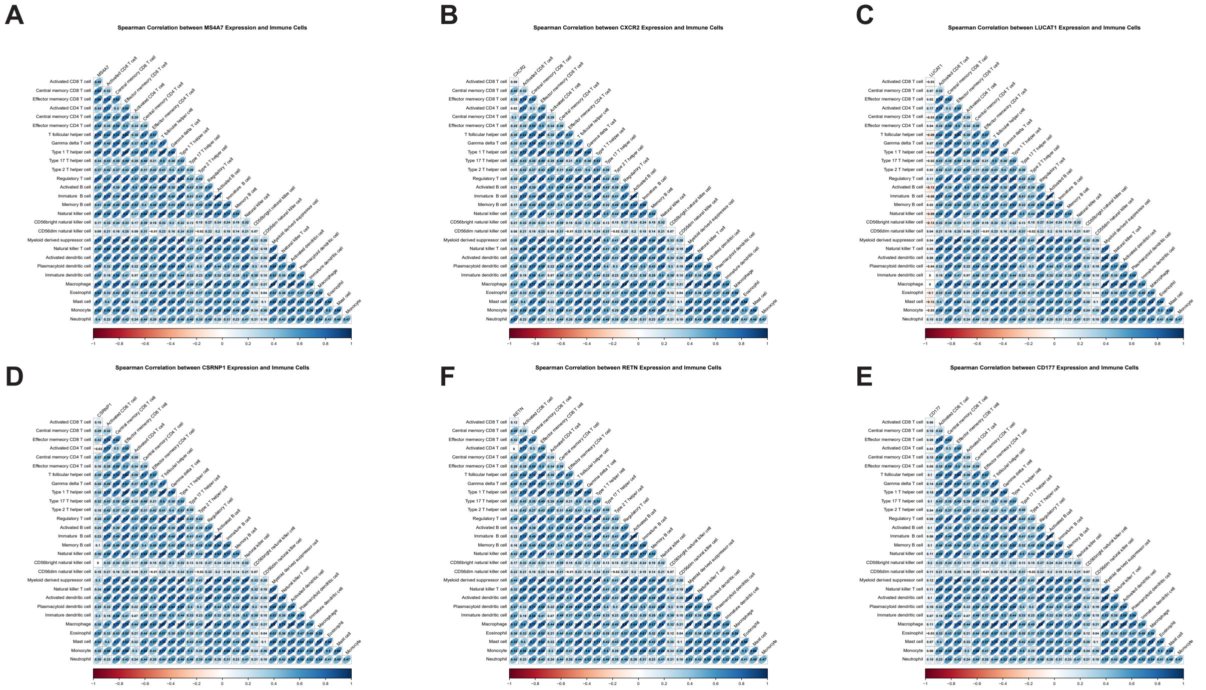
A
Spearman Correlation between MS4A7 Expression and Immune Cells
B
Spearman Correlation between CXCR2 Expression and Immune Cells
C
Spearman Correlation between LUCAT1 Expression and Immune Cells
D
Spearman Correlation between CSRNP1 Expression and Immune Cells
F
Spearman Correlation between RETN Expression and Immune Cells
E
Spearman Correlation between CD177 Expression and Immune Cells
